# Supplementary material for: HIVprotI: an integrated web based platform for prediction and design of HIV proteins inhibitors
Source: J Cheminform. 2018 Mar 9;10:12. doi: 10.1186/s13321-018-0266-y (PMC5845081; doi:10.1186/s13321-018-0266-y)
Supplement: Supplementary file 2 — Additional file 2. Source code of HIVProtI web server. [file 13321_2018_266_MOESM2_ESM.zip › HIVprotI_Source-code/hivprotI/weka-3-6-6/doc/weka/classifiers/bayes/blr/LaplacePriorImpl.html]

LaplacePriorImpl


---


|  |  |  |
| --- | --- | --- |
| **Overview** \| **Package** \| **Class** \| **Tree** \| **Deprecated** \| **Index** \| **Help** \| **Weka's home** | |  |
| **PREV CLASS**   **NEXT CLASS** | **FRAMES**    **NO FRAMES**     **All Classes** |  |
| SUMMARY: NESTED \| FIELD \| CONSTR \| METHOD | DETAIL: FIELD \| CONSTR \| METHOD |  |


---


## weka.classifiers.bayes.blr Class LaplacePriorImpl

```
java.lang.Object
  weka.classifiers.bayes.blr.Prior
      weka.classifiers.bayes.blr.LaplacePriorImpl
```

**All Implemented Interfaces:**: java.io.Serializable, RevisionHandler

---

``` public class LaplacePriorImpl extends Prior ```

Implementation of the Gaussian Prior update function based on modified
CLG Algorithm (CLG-Lasso) with a certain Trust Region Update based
on Laplace Priors.

**Version:**
:   $Revision: 1.2 $

**Author:**
:   Navendu Garg(gargnav@iit.edu)

**See Also:**: Serialized Form

---

**Constructor Summary**

`LaplacePriorImpl()`


| **Method Summary** | |
| --- | --- |
| `void` | `computeLogLikelihood(double[] betas, Instances instances)`             Computes the log-likelihood values using the implementation in the Prior class. |
| `void` | `computePenalty(double[] betas, double[] hyperparameters)`             This function computes the penalty term specific to Laplacian distribution. |
| `java.lang.String` | `getRevision()`             Returns the revision string. |
| `double` | `laplaceUpdate(int j, double sign)`             This is the CLG-lasso update function described in the |
| `double` | `update(int j, Instances instances, double beta, double hyperparameter, double[] r, double deltaV)`             Update function specific to Laplace Prior. |

**Methods inherited from class weka.classifiers.bayes.blr.Prior**

`computelogLikelihood, getLoglikelihood, getLogPosterior, getPenalty`

**Methods inherited from class java.lang.Object**

`equals, getClass, hashCode, notify, notifyAll, toString, wait, wait, wait`

**Constructor Detail**

### LaplacePriorImpl

```
public LaplacePriorImpl()
```


**Method Detail**

### update

```
public double update(int j,
                     Instances instances,
                     double beta,
                     double hyperparameter,
                     double[] r,
                     double deltaV)
```

:   Update function specific to Laplace Prior.

    :   **Overrides:**: `update` in class `Prior`

---


### laplaceUpdate

```
public double laplaceUpdate(int j,
                            double sign)
```

:   This is the CLG-lasso update function described in the

    ```
     @TechReport{blrtext04,
    author = {Alexander Genkin and David D. Lewis and David Madigan},
    title = {Large-scale bayesian logistic regression for text categorization},
    institution = {DIMACS},
    year = {2004},
    url = "http://www.stat.rutgers.edu/~madigan/PAPERS/shortFat-v3a.pdf",
    OPTannote = {}
    }
    ```

    :   **Parameters:**: `j` - **Returns:**: double value

---


### computeLogLikelihood

```
public void computeLogLikelihood(double[] betas,
                                 Instances instances)
```

:   Computes the log-likelihood values using the implementation in the Prior class.

    :   **Parameters:**: `betas` -: `instances` -: `hyperparameter` -

---


### computePenalty

```
public void computePenalty(double[] betas,
                           double[] hyperparameters)
```

:   This function computes the penalty term specific to Laplacian distribution.

    :   **Overrides:**: `computePenalty` in class `Prior`
    :   **Parameters:**: `betas` -: `hyperparameters` -

---


### getRevision

```
public java.lang.String getRevision()
```

:   Returns the revision string.

    :   **Returns:**: the revision


---


|  |  |  |
| --- | --- | --- |
| **Overview** \| **Package** \| **Class** \| **Tree** \| **Deprecated** \| **Index** \| **Help** \| **Weka's home** | |  |
| **PREV CLASS**   **NEXT CLASS** | **FRAMES**    **NO FRAMES**     **All Classes** |  |
| SUMMARY: NESTED \| FIELD \| CONSTR \| METHOD | DETAIL: FIELD \| CONSTR \| METHOD |  |


---
